# Preserving Nasal Tip Rotation and Projection in Open Septorhinoplasty

**DOI:** 10.31486/toj.22.0006

**Published:** 2022

**Authors:** Jason D. Pou, John Ziegler, Krishna G. Patel, Samuel L. Oyer

**Affiliations:** ^1^ Department of Otolaryngology, Head and Neck Surgery, Ochsner Clinic Foundation, New Orleans, LA; ^2^ Department of Otolaryngology, Head and Neck Surgery, Medical University of South Carolina, Charleston, SC; ^3^ Department of Otolaryngology, Head and Neck Surgery, University of Virginia Medical Center, Charlottesville, VA

**Keywords:** *Nasal septum*, *nasal surgical procedures*, *projection*, *rhinoplasty*, *rotation*

## Abstract

**Background:** Open septorhinoplasty is a common facial plastic surgery procedure that requires extensive planning and knowledge to achieve predictable outcomes. Many patients want to keep their nasal tip characteristics, and the surgeon's task is to reliably meet this expectation and provide stable long-term results. Techniques used to reconstruct nasal tip support include the tongue-in-groove, caudal septal extension graft, and caudal septal replacement graft procedures.

**Methods:** We assessed the 1-year reliability of tongue-in-groove, caudal septal extension graft, and caudal septal replacement graft procedures in maintaining nasal tip rotation and projection in open septorhinoplasty. We conducted a retrospective case series review of septorhinoplasty cases between 2015 and 2019 at the Medical University of South Carolina. Cases with intention to change nasal tip rotation or projection were excluded. Two blinded reviewers analyzed standardized preoperative and 1-year postoperative photographs.

**Results:** Fifty-seven patients fit the inclusion criteria and were included in the analysis. Mean preoperative and postoperative nasal tip rotations and projection ratios were similar (*P*=0.62, *P*=0.22, respectively). Twenty-six patients underwent a tongue-in-groove procedure, 24 had a caudal septal extension graft, and 7 had a caudal septal replacement graft with preoperative nasal tip rotations of 98.93°, 99.35°, and 96.89°, respectively (*P*=0.73). At 1 year, patients who received a tongue-in-groove procedure had a significant increase in nasal tip rotation to 101.24° (*P*=0.013), while patients who received a caudal septal extension graft had a significant decrease in nasal tip rotation to 97.25° (*P*=0.009). Patients who received a caudal septal replacement graft had no significant change in nasal tip rotation (*P*=0.117). The preoperative and postoperative projection ratios were not significantly different among the 3 techniques.

**Conclusion:** Tongue-in-groove, caudal septal extension graft, and caudal septal replacement graft are reliable techniques for maintaining nasal tip projection in open septorhinoplasty. In our experience, when attempting to maintain preoperative nasal tip rotation, the tongue-in-groove technique resulted in a significant increase in tip rotation of 2.31°, while the caudal septal extension graft resulted in a significant decrease of 2.1° at 1 year postoperatively.

## INTRODUCTION

Open septorhinoplasty is a common facial plastic surgery procedure that requires extensive planning and knowledge to achieve predictable outcomes. The open septoplasty approach is frequently required for severe septal deviations, caudal deformities, and significantly crooked noses and involves dividing the interdomal ligament to expose the septum. One key step in the procedure is restoring nasal tip rotation and projection. Postoperative loss of nasal tip support is one of the most common faults in septorhinoplasty.^1^

Controlling nasal tip rotation is a complex aspect of rhinoplasty. Nasal tip rotation, generally, is the relationship between the base of the nose and the face in profile view. Rotation can be determined in 5 ways: the nasolabial angle, the columellar-facial angle, the nostril axis angle compared to the facial plane, the nostril axis angle compared to a line perpendicular to the Frankford horizontal line, and the columellar-facial angle in relation to a line perpendicular to the Frankfurt horizontal line.^2^ Many patients want to keep their nasal tip characteristics, and the surgeon's task is to reliably meet this expectation and provide stable long-term results.

Techniques used to reconstruct nasal tip support include the tongue-in-groove technique and the caudal septal extension graft. The caudal septal replacement graft is a technique used for a severely deviated or fractured caudal septum in which the native caudal strut cannot be salvaged.

Tongue-in-groove, a common technique used to reconstruct the nasal tip, was originally described for the treatment of an elongated nose by Rethi in 1934.^3^ Evolution of the tongue-in-groove technique has proven successful in modulating nasal tip rotation and projection, as well as treating alar-columellar disharmony.^4^ For the tongue-in-groove technique, the medial crura are advanced cephaloposteriorly onto the caudal septum; this procedure can be performed in both open and endonasal rhinoplasty.^5^

Byrd et al first described the caudal septal extension graft in 1997, and this technique has been used to control nasal length and tip rotation, projection, and shape.^6^ The medial crura are fashioned similarly to the tongue-in-groove technique, but they are secured to a cartilage graft fixed to the caudal septum.

Foda introduced the caudal septal replacement graft in 2008 for restoring nasal tip support in cases with caudal septal deficiency.^7^ The caudal septal replacement graft is used when significant caudal septal deficiency or deformity requires complete replacement of the caudal strut.

Deciding which technique to perform requires an understanding of the patient's anatomy and the long-term changes that occur over months to years. This study was designed to measure and compare the 1-year changes in nasal tip rotation and projection after tongue-in-groove, caudal septal extension graft, and caudal septal replacement graft open septorhinoplasty.

## METHODS

We retrospectively reviewed all open septorhinoplasty procedures performed at the Medical University of South Carolina by the Facial Plastic and Reconstructive Surgery Division from 2015 to 2019 (251 total patients). Patients were excluded if an open septoplasty approach was not performed, if they did not have a 12-month postoperative follow-up visit with standardized photographs, or if intervention or revision surgery took place within the 1-year postoperative period. Seventy-two patients underwent open septorhinoplasty and had 12-month postoperative photos. After excluding cases with intention to change nasal tip rotation and cases with radix grafts or chin augmentation, a total of 57 patients were included for analysis. For these patients, the following data were extracted from the medical record: patient age at time of surgery, sex, history of trauma, prior septal surgery, and technique used to reset the nasal tip. The study was approved by the institutional review board of the senior author's primary institution.

**Figure 1.**
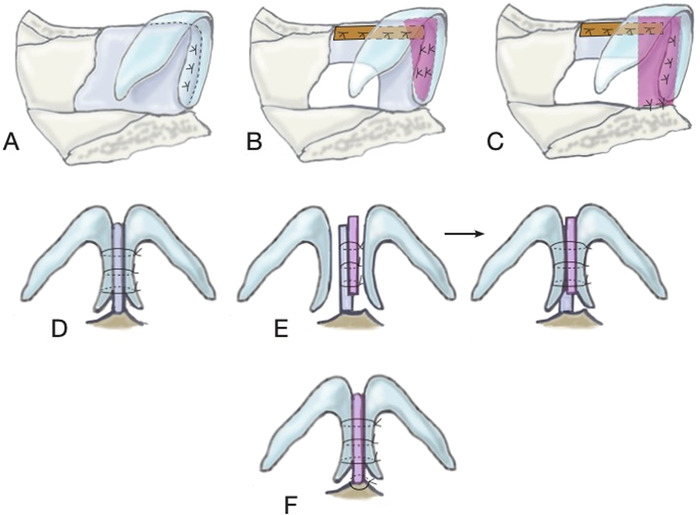
**(A) Tongue-in-groove technique is demonstrated in lateral view with medial footplates sutured to the caudal edge of the septum. (B) Caudal septal extension graft is demonstrated in lateral view with extended spreader graft (orange) and caudal septal extension graft (pink) with medial footplates sutured to the caudal septal extension graft. (C) Caudal septal replacement graft is demonstrated in lateral view with caudal septal replacement graft (pink) sutured to the nasal spine and dorsal septum/extended spreader graft (degree of dorsal septal removal varies based on the extent of deviation). (D) Tongue-in-groove technique is demonstrated in base view. (E) Caudal septal extension graft is demonstrated in base view with side-to-side caudal septal extension graft (pink). (F) Caudal septal replacement graft is demonstrated in base view with suture attachments to the nasal spine.** (Graphic by Barbara Siede, Ochsner Clinic Foundation. For readers of the print publication, a color version of this figure is available at https://doi.org/10.31486/toj.22.0006.)

Absorbable 5-0 PDS sutures were used to reset the tip for all 3 techniques (Figure 1). In most cases, the caudal septal extension graft was performed with septal cartilage in a side-to-side technique, and the caudal septal replacement graft was performed with costal cartilage.

**Figure 2.**
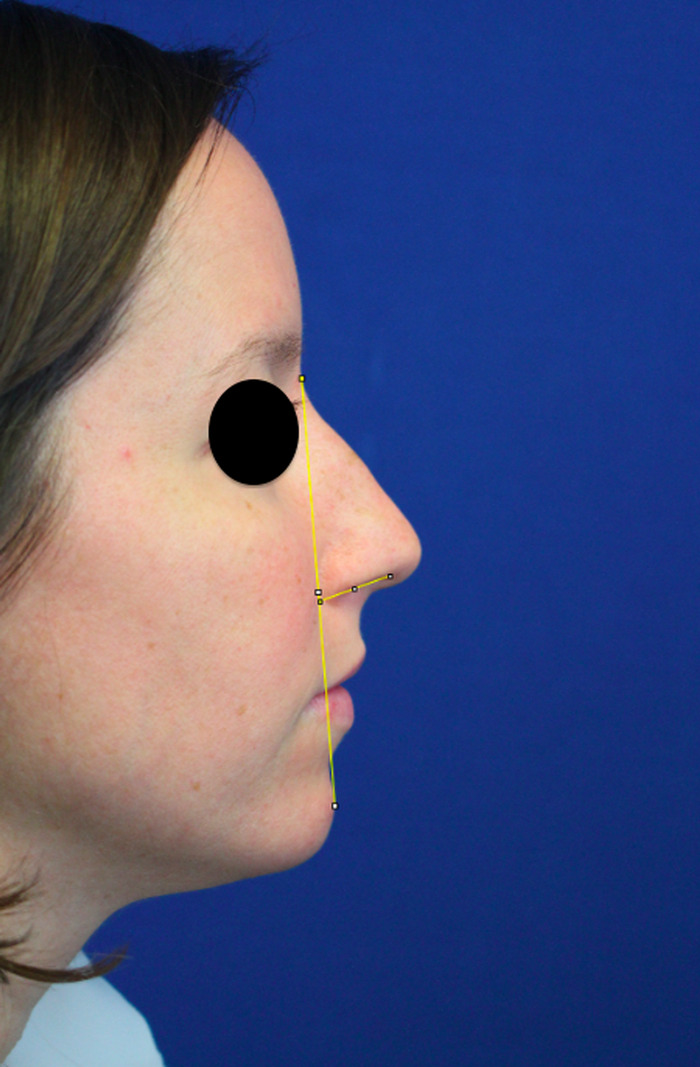
Nasal tip rotation was measured using the facial plane and the nostril axis.

**Figure 3.**
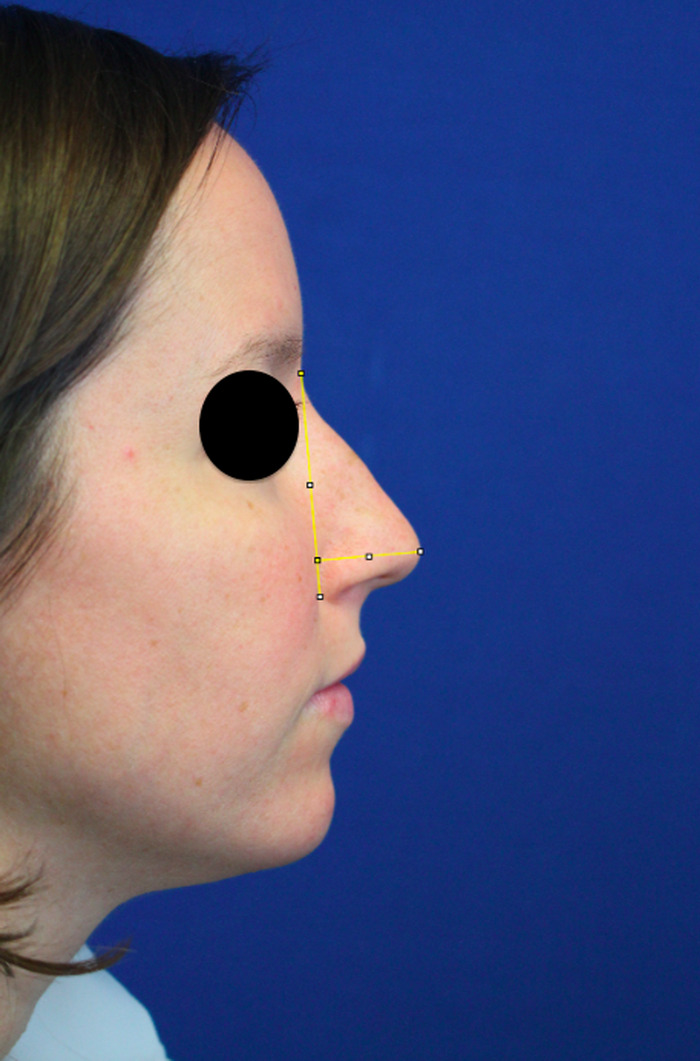
Nasal tip projection was measured using the ratio of projection (tip-defining point to alar-facial groove) to the nasal height (sellion to inferior-most point of the alar-facial groove).

Two blinded reviewers systematically analyzed the preoperative and postoperative photographs for all patients in the study. The average of the reviewers’ scores was recorded. Nasal tip rotation was analyzed using the nostril axis (line from the most anterior and most posterior point of the nostril) in relation to the facial plane (line from the sellion to the soft tissue pogonion) (Figure 2). The angle was measured using the ImageJ computer software (National Institutes of Health) protractor feature.^8^ Projection was analyzed by measuring the distance from the tip-defining point to the alar-facial groove using a line perpendicular to the facial plane. Although standardized photography was used, minor zoom adjustments required projection to be measured with a ratio. The ratio of projection to nasal height (distance from the sellion to the inferior-most point of the alar-facial groove) was used (Figure 3).

Preoperative nasal tip rotations and projections were compared using analysis of variance 1-way tests. The 1-year postoperative nasal tip rotations and projections for the 3 techniques were also compared with analysis of variance 3-way tests. Preoperative and 1-year postoperative nasal tip rotations and projections were compared using paired *t* tests. *P* values <0.05 were considered significant. Statistical analysis was completed using SAS, version 9.3 (SAS Institute Inc.).

## RESULTS

For all 57 patients included in the analysis, the average preoperative and postoperative nasal tip rotations were 98.86° and 99.5° (*P*=0.62), respectively, and the average preoperative and postoperative projection ratios were 0.55 and 0.56 (*P*=0.22), respectively (Table 1). Thirty-three patients were female, and 24 were male. The preoperative and postoperative projection ratios for males were significantly higher compared to those for females (*P*=0.002 and *P*=0.048, respectively). Females had a significant increase in projection ratios at 1 year postoperatively (*P*=0.02). Patients undergoing revision septorhinoplasty had significantly decreased preoperative projection ratios compared to primary cases (*P*=0.018), but no significant difference was observed postoperatively. Neither age nor history of trauma had a significant correlation with nasal tip rotation or projection ratio.

**Table 1.** Preoperative and Postoperative Patient Characteristics

Variable	n	Preoperative Rotation, Degrees, Mean ± SD	Postoperative Rotation, Degrees, Mean ± SD	*P* Value	Preoperative Projection Ratio	Postoperative Projection Ratio	*P* Value
All patients	57	98.86 ± 7.12	99.5 ± 6.9	0.62	0.55	0.56	0.22
Female	33	100.11 ± 6.97	100.63 ± 7.42	0.49	0.53	0.55	**0.02**
Male	24	97.15 ± 7.12	97.97 ± 5.92	0.47	0.59	0.58	0.64
*P* value		0.12	0.15		**0.002**	**0.048**	
≤35 years	30	100.1 ± 5.92	100.03 ± 6.65	0.93	0.56	0.57	0.27
>35 years	27	97.49 ± 8.15	98.93 ± 7.25	0.16	0.54	0.55	0.48
*P* value		0.17	0.55		0.34	0.28	
Primary rhinoplasty	35	99.14 ± 5.94	99.68 ± 6.37	0.48	0.57	0.57	0.93
Revision rhinoplasty	22	98.39 ± 8.94	99.22 ± 7.88	0.49	0.53	0.55	0.073
*P* value		0.71	0.81		**0.018**	0.17	
Trauma	35	98.26 ± 7.98	99.61 ± 6.18	0.1	0.56	0.56	0.8
No trauma	22	99.83 ± 5.53	99.34 ± 8.07	0.64	0.54	0.56	0.06
*P* value		0.42	0.69		0.43	0.69	

Note: Significant *P* values are highlighted in bold.

Twenty-six patients underwent a tongue-in-groove procedure, 24 had a caudal septal extension graft, and 7 had a caudal septal replacement graft with preoperative nasal tip rotations of 98.93°, 99.35°, and 96.89°, respectively (*P*=0.73). Patients who underwent a tongue-in-groove procedure had a significant increase in nasal tip rotation to 101.24° (*P*=0.013) at 1 year postoperatively, while patients who had a caudal septal extension graft had a significant decrease in nasal tip rotation to 97.25° (*P*=0.009) at 1 year (Table 2). Although we saw a trend toward increased nasal tip rotation (from 96.89° to 100.8°) in the patients receiving a caudal septal replacement graft, this difference was not significant (*P*=0.117). Preoperative and postoperative projection ratios were not significantly different among the 3 techniques.

**Table 2.** Nasal Tip Rotation and Projection Comparison of Tongue-in-Groove (TIG), Caudal Septal Extension Graft (CSEG), and Caudal Septal Replacement Graft (CSRG) Procedures

Procedure	n	Preoperative Rotation, Degrees, Mean ± SD	Postoperative Rotation, Degrees, Mean ± SD	*P* Value	Preoperative Projection Ratio	Postoperative Projection Ratio	*P* Value
TIG	26	98.93 ± 7.29	101.24 ± 7.54	**0.013**	0.55	0.56	0.52
CSEG	24	99.35 ± 6.56	97.25 ± 6.23	**0.009**	0.55	0.57	0.155
CSRG	7	96.89 ± 9.05	100.8 ± 4.86	0.117	0.56	0.55	0.56
*P* value		0.73	0.11		0.9	0.81	

Note: Significant *P* values are highlighted in bold.

In the 24 patients who underwent a caudal septal extension graft, septal cartilage was used in 18 cases, costal cartilage in 3, auricular cartilage in 2, and cadaveric cartilage in 1 case. Although we found a significant decrease in postoperative nasal tip rotation compared to the preoperative rotation with septal cartilage (2.02°, *P*=0.032) and costal cartilage (4.88°, *P*=0.02), the preoperative and postoperative nasal tip rotation and projection ratios were not significantly different between the septal and costal cartilage graft groups (Table 3).

**Table 3.** Nasal Tip Rotation and Projection Comparison of Caudal Septal Extension Graft (CSEG) and Caudal Septal Replacement Graft (CSRG) Cartilage Types

Procedure/Cartilage Type	n	Preoperative Rotation, Degrees, Mean	Postoperative Rotation, Degrees, Mean	*P* Value	Preoperative Projection Ratio	Postoperative Projection Ratio	*P* Value
CSEG							
Septal	18	98.87	96.85	**0.032**	0.57	0.57	0.46
Costal	3	103.03	98.15	**0.02**	0.49	0.55	0.22
*P* value		0.35	0.76		0.08	0.6	
CSRG							
Costal	4	101.1	103.8	0.59	0.55	0.55	0.88
Cadaveric	2	85.6	95.4	0.32	0.59	0.53	0.5
*P* value		**0.036**	0.11		0.4	0.71	

Note: Significant *P* values are highlighted in bold.

In the 7 patients who underwent a caudal septal replacement graft, costal cartilage was used in 4 cases, cadaveric cartilage was used in 2, and septal cartilage was used in 1 case. Although preoperative nasal tip rotation was significantly higher in patients who had costal vs cadaveric caudal septal replacement graft (101.1° vs 85.6°, *P*=0.036), postoperative nasal tip rotation and preoperative and postoperative projection ratios were not significantly different between the 2 groups.

## DISCUSSION

Control and long-term predictability of the nasal tip position are crucial for favorable rhinoplasty results. Because the literature is limited, the goal of this study was to compare 1-year nasal tip rotation and projection between tongue-in-groove, caudal septal extension graft, and caudal septal replacement graft techniques for patients who wished to maintain their preoperative nasal tip position.^2,9,10^ While these techniques can be used to modulate nasal tip rotation and projection, they are often used to restore the nasal tip when the open septoplasty approach is performed. We specifically analyzed cases in which the goal was to restore preoperative nasal tip rotation and projection after correcting the underlying septal and nasal valve deformities.

The tongue-in-groove technique is a well-known maneuver in rhinoplasty to control nasal tip rotation and projection and improve nasal tip support. Antunes and Quatela demonstrated a significant loss of nasal tip rotation with the tongue-in-groove technique during the first postoperative year.^2^ This loss was hypothesized to be attributable to the gravitational forces on the nasal tip, as well as the intrinsic resiliency of the lateral crura. Postoperative nasal tip rotation decreased by an average of 7.9° after 1 year; thus, overcorrection is typically desired.^2^ Kadakia and Ovchinsky compared absorbable vs nonabsorbable sutures for the tongue-in-groove technique in endonasal rhinoplasty.^4^ They demonstrated a significant increase in nasal tip rotation at 1 year with permanent sutures that was not present with absorbable PDS sutures.^4^ In our practice, we use 5-0 PDS sutures for the tongue-in-groove technique, with over-rotation of approximately 8° to 10°.

The caudal septal extension graft is commonly used in rhinoplasty to control nasal length as well as nasal tip rotation and projection. The caudal septal extension graft lengthens the caudal septum and thus allows for a decrease in nasal tip rotation when needed. The caudal septal extension graft has been found to have more stability in nasal tip rotation over time than columellar strut grafts. Although Sawh-Martinez et al demonstrated a trend toward a decrease in nasal tip rotation over time with the caudal septal extension graft vs the columellar strut graft, they found no significant difference between 6-week and 12-month postoperative nasal tip rotation.^9^ In our study, caudal septal extension grafts were used when the caudal septum was judged to be deficient to maintain the patient's preoperative nasal tip rotation. We did not measure immediate postoperative nasal tip rotation, but 12-month postoperative rotation was significantly decreased with the caudal septal extension graft technique.

Patients having the tongue-in-groove procedure had a significant increase in nasal tip rotation of 2.31° at 12 months postoperatively. This increase may be the result of too much overcorrection during the initial maneuver or overutilization of this technique when increased length of the caudal septum is needed. Measurements were performed 1 year postoperatively, so a decrease in nasal tip rotation may possibly occur with time. Conversely, patients having the caudal septal extension graft procedure had a significant decrease in nasal tip rotation of 2.1°. This change is likely attributable to a loss in nasal tip rotation over time because of the same gravitational forces that affect rotation in tongue-in-groove procedures. Absorbable PDS sutures are used for both techniques in our practice; thus, tongue-in-groove and caudal septal extension graft ultimately rely on scarring and fibrosis to maintain nasal tip position. While 1 year is a commonly referenced postoperative timepoint in rhinoplasty, continued changes may still be possible over decades, and studies are necessary to determine the true durability of tongue-in-groove and caudal septal extension graft results.

Our study population included few caudal septal replacement graft cases. When replacing the entire caudal septum, additional variables must be considered that are not present with tongue-in-groove and caudal septal extension graft procedures. The caudal septal replacement graft must be secured to the nasal spine and crest, as well as the dorsal septum, and is typically performed with extended spreader grafts in our practice. The degree and location of the caudal septal deviation determine the extent of septal cartilage that is removed and replaced. Although studies have demonstrated no increased risk of resorption with cadaveric cartilage, in our opinion, costal or septal cartilage is ideal for the caudal septal replacement graft because of improved stability.^10^ The 2 patients in our study who received caudal septal replacement grafts with cadaveric cartilage had no adverse events or significant differences in postoperative nasal tip rotation or projection ratios when compared to patients who received costal cartilage.

For restoring the nasal tip in open septorhinoplasty, we feel that the tongue-in-groove technique is appropriate for patients with adequate caudal septal length and height and those who can tolerate a slight increase in rotation. Rhinoplasty surgeons typically overcorrect with the tongue-in-groove technique because some loss of nasal tip rotation is expected with time.^2^ Suture position along the vertical axis of the septum allows adjustment in nasal tip rotation as the tip is restored.^11^ The caudal septal extension graft is used for increasing nasal tip support in patients with insufficient caudal septal cartilage and/or patients who cannot tolerate an increase in tip rotation. The surgeon should consider slight overcorrection of 2° to 3° with the caudal septal extension graft as it experiences the same gravitational forces as the tongue-in-groove technique. Although we identified a significant change in nasal tip rotation with these techniques, clinically, the changes were small and none of the patients in the study were dissatisfied by the change in rotation.

Projection ratio stability was maintained with all 3 techniques used in the study. When restoring the nasal tip in open septorhinoplasty, suture position along the horizontal axis of the septum can be adjusted to modulate nasal tip projection without changing the rotation.^10^ A stable foundation without horizontal mobility is critical with the caudal septum or caudal septal replacement graft securely fixed to the spine. In our practice, we use at least 2 sutures spaced approximately 1 cm apart along the nasal spine and maxillary crest when resetting or replacing the caudal septum. In our experience, the tongue-in-groove, caudal septal extension graft, and caudal septal replacement graft are all reliable techniques for maintaining nasal tip projection at 1 year postoperatively.

This study has several limitations. Although all cases were performed with the goal of maintaining the patients’ preoperative nasal tip rotations and projections, patient characteristics may have created some bias with the surgeons’ choice of tip reconstruction. Second, immediate postoperative nasal tip rotation and projection were not recorded, and follow-up was only measured for 1 year. Consequently, we could not evaluate changes that happened very early or very late in the postoperative course.

## CONCLUSION

Tongue-in-groove, caudal septal extension graft, and caudal septal replacement graft are reliable techniques for maintaining nasal tip projection in open septorhinoplasty. In our experience, despite attempting to maintain preoperative nasal tip rotation, the tongue-in-groove technique resulted in a significant increase in tip rotation of 2.31°, the caudal septal extension graft resulted in a significant decrease of 2.1°, and no change was seen with caudal septal replacement graft 1 year postoperatively. Studies assessing immediate and long-term postoperative rotation are necessary to determine the long-term effect of gravity on these rhinoplasty techniques.

## Notes

*This article meets the Accreditation Council for Graduate Medical Education and the American Board of Medical Specialties Maintenance of Certification competencies for Patient Care and Medical Knowledge.*
